# Efficacy and Safety of an mHealth App and Wearable Device in Physical Performance for Patients With Hepatocellular Carcinoma: Development and Usability Study

**DOI:** 10.2196/14435

**Published:** 2020-03-11

**Authors:** Yoon Kim, Jinserk Seo, So-Yeon An, Dong Hyun Sinn, Ji Hye Hwang

**Affiliations:** 1 Department of Physical and Rehabilitation Medicine Samsung Medical Center Sungkyunkwan University School of Medicine Seoul Republic of Korea; 2 Department of Rehabilitation Medicine Incheon St. Mary's Hospital The Catholic University of Korea Incheon Republic of Korea; 3 Department of Health Science Korea University Graduate School Korea University Seoul Republic of Korea; 4 Department of Internal Medicine Samsung Medical Center Sungkyunkwan University School of Medicine Seoul Republic of Korea

**Keywords:** mHealth, hepatocellular carcinoma, rehabilitation, exercise, physical fitness, physical activity

## Abstract

**Background:**

Exercise is predicted to have a positive effect among hepatocellular carcinoma (HCC) patients. However, these patients are hesitant to start and build up an exercise program for one major reason: the vague fear of developing hepatic decompensation, a potentially fatal condition that can lead to death. Integrating mobile health (mHealth) with individualized exercise programs could be a possible option for promoting physical capacity among HCC patients.

**Objective:**

The aim of this study was to evaluate the efficacy and safety of rehabilitation exercises, which have been individually prescribed via an mHealth app, on physical fitness, body composition, biochemical profile, and quality of life among HCC patients.

**Methods:**

A total of 37 HCC patients were enrolled in a 12-week course with an mHealth app program targeted to HCC patients. The wearable wristband device Neofit (Partron Co) was provided to participants, and recorded daily physical data, such as the number of steps, calorie expenditure, exercise time, and heart rate. Each participant was given an individualized rehabilitation exercise program that was prescribed and adjusted at the 6-week midintervention period based on the assessment results. At baseline, 6-week, and 12-week sessions, participants’ physical fitness levels (ie, 6-minute walk test, grip strength test, and 30-second chair stand test) were measured. Physical activity levels, as measured by the International Physical Activity Questionnaire-Short Form (IPAQ-SF); body composition (ie, body mass index, body fat percentage, and muscle mass); biochemical profiles; and quality of life, as measured by the European Organization for Research and Treatment of Cancer Quality-of-Life Questionnaire C30, were assessed at baseline and at the end point. At the 6-week midpoint, exercise intensity was individually adjusted.

**Results:**

Of the 37 patients, 31 (84%) completed the 12-week intervention. Grip strength improved significantly after 12 weeks of the intervention. The 30-second chair stand test and the 6-minute walk test showed significant improvement from 0 to 6 weeks, from 0 to 12 weeks, and from 6 to 12 weeks. Muscle mass and the IPAQ-SF score increased significantly after 12 weeks of the intervention without biochemical deterioration.

**Conclusions:**

Following 12 weeks of mHealth care, including an individually prescribed rehabilitation exercise program, we saw significant improvements in physical fitness, body composition, and physical activity without any complication or biochemical deterioration among compensated HCC patients who had completed therapy.

## Introduction

Physical activity has been proven to have a positive influence, both biologically and functionally, in patients with cancer, such as breast or prostate cancer [1-3]. Recent studies indicate that even patients undergoing acute cancer treatments can benefit from individualized exercise programs [4]. However, little is known about the role of exercise on hepatocellular carcinoma (HCC), which is the fifth- and seventh-most common cancer worldwide in men and women, respectively [5].

Among the few studies regarding the effect of exercise in HCC, one study reported that continuous regular exercise improved physical ability without deteriorating liver function in HCC patients with chronic liver disease (CLD) [6]. An experimental study using a rat model found that regular physical activity reduced the risk of the primary development of HCC [7]. Although the positive effect of exercise is quite predictable in HCC patients, these patients are hesitant to start and build up an exercise program for one major reason: the vague fear of developing hepatic decompensation. Hepatic decompensation is a potentially fatal condition that can be encountered by patients with HCC, which includes hepatic encephalopathy, esophageal varices, or ascites. Therefore, in order to safely boost exercise capacity, a delicate supporting system is needed to monitor physical activity and alert such vulnerable patients before hepatic decompensation occurs.

Integrating mobile health (mHealth) with individualized exercise programs could be a possible option for promoting physical capacity of HCC patients. Following the explosive increase in smartphone penetration globally, mHealth has been spotlighted as a novel technology for promoting exercise programs, not only among healthy populations but also among patients with various diseases, such as diabetes, heart disease, and cancer [8-10]. Several recent studies have reported the effectiveness of mHealth in patients with solid cancers and survivors of breast cancer and colorectal cancers [4,11]. mHealth promotes physical activity among cancer patients by motivating them to exercise and by providing real-time feedback [12,13]. Because mHealth can provide large amounts of information and even lower the barrier of communication with health care providers, it is an ideal tool for helping HCC patients to exercise safely outside the hospital.

In this study, we evaluated the efficacy and safety of exercises that were individually prescribed to compensated HCC patients after anticancer therapy. We analyzed changes in physical fitness, body composition, biochemical profile, and quality of life (QoL) after 12 weeks of an mHealth exercise intervention.

## Methods

### Participants

HCC patients who visited the outpatient cancer rehabilitation clinic of a tertiary hospital from November 2017 to February 2018 were prospectively enrolled in this study. The inclusion criteria were as follows: HCC patients aged 19-69 years, patients at stage I or II of the modified Union for International Cancer Control (mUICC) staging system, patients with a Child-Pugh class A or B score, patients who could walk independently for 30 minutes, and patients who had a mobile phone. Exclusion criteria were as follows: patients who required exercise restrictions for severe cardiopulmonary or renal disease, patients with musculoskeletal or neurological deficits, patients with cognitive impairments that interfered with mobile phone utilization, or patients who were unable to give written consent.

### App Development

**Figure 1:**
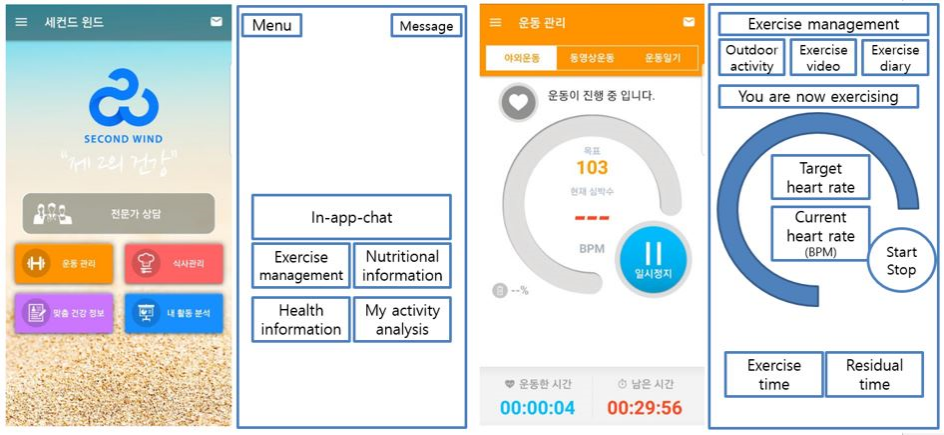
App screenshots and configuration. Screenshots of the main page (far left) and exercise management page (middle right) are shown, along with respective diagrams of explanations to the right of each screenshot.

Based on our previous experience of, and knowledge about, developing mHealth apps for specific cancer patients, we recruited app engineers and five health care professionals from a comprehensive cancer center [4]. Health care professionals were recruited to help in the development of a comprehensive mHealth care system tailored to HCC patients, in order to provide health information, self-monitoring, and connections with health care professionals. The app included the following features: *exercise management*, *nutritional information*, *health information*, *my activity analysis*, and *in-app chat* service (see Figure 1).

The exercise management feature provided daily, personalized aerobic and anaerobic exercises based on clinical evidence. The health information content changed daily, including general health information about HCC, medication, adverse effects of anticancer therapy, and nutrition. Real-time communication with a medical professional was available through the in-app chat service. The wearable Internet of Things (IoT) device, which connected with the mobile app, gathered real-time physical data to monitor both physical activity and the health of the participants. A clinical evidence-based care system with the above functions was implemented through the mHealth app.

### Intervention

#### mHealth Care App

All participants received a 12-week course of an individually prescribed exercise program through the mHealth app using their own mobile phones and an interconnected wearable IoT device worn on their wrists. The participants were told to download the mobile app to their mobile phones. The mobile app consisted of general medical information on HCC, disease-specific exercise care, nutritional information, and a real-time chat service that directly connected participants to the study coordinator. The wearable IoT device—Neofit (Partron Co)—which we provided to the participants, recorded daily physical data, such as the number of steps, calorie expenditure, exercise time, and heart rate. Each participant was given an individualized rehabilitation exercise program that was prescribed and adjusted at the 6-week midintervention period based on the assessment results.

#### Individually Prescribed Rehabilitation Exercises

**Figure 2:**
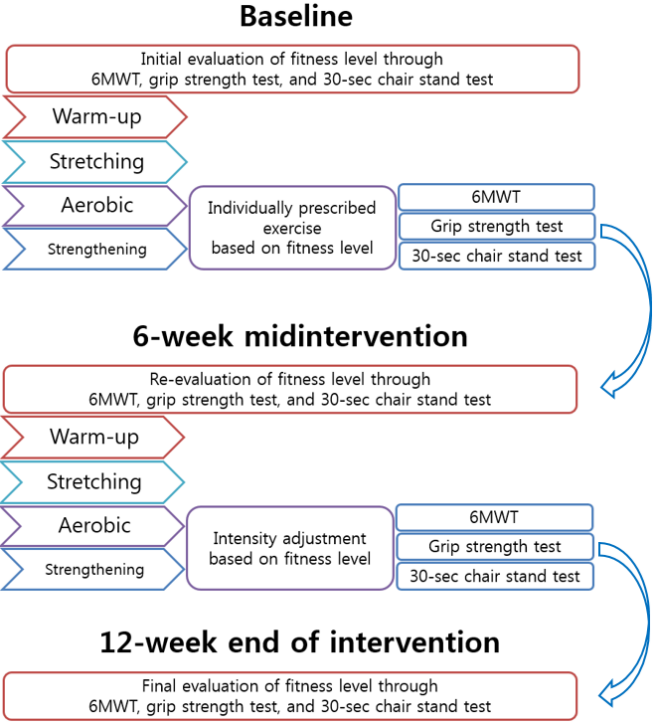
Procedure for adjusting participants' individually prescribed rehabilitation exercise programs in the mHealth app. 6MWT: 6-minute walk test.

The patients were recommended to participate in individually tailored regular rehabilitation exercises. Video clips were provided by the app and were composed of warm-up, stretching, aerobic, and muscle-strengthening exercises for the upper and lower extremities. Participants were asked to watch the video clips and perform the exercises daily. During the baseline assessment, participants received instructions for standardized warm-up and stretching exercises and individualized aerobic and muscle-strengthening exercises. The aerobic and muscle-strengthening exercise prescriptions were modified based on each participant’s altered physical fitness levels measured at the 6-week midpoint session (see Figure 2).

The intensity (ie, light walking, light running, mountain climbing, and cycling) and target heart rate for the aerobic exercise were set from the results of the 6-minute walk test (6MWT). This result was compared with an individualized reference value calculated using each participant’s age, height, and weight [14]. If the 6MWT result was higher than the individualized reference value, the recommended exercise intensity and target heart rate were increased.

All the major muscle groups of the upper extremities, lower extremities, and trunk were included in the muscle-strengthening exercise program. Three steps of resistance exercises (ie, maximum, moderate, and minimum resistance) were provided. The results of the 30-second chair stand test and grip strength test were compared with reference values based on the healthy, normal, Korean population from the Korea Sports Promotion Foundation. Appropriate intensity levels for the strengthening exercises were determined according to those results.

After 6 weeks of participation, fitness levels (ie, 6MWT, grip strength test, and 30-second chair stand test) were assessed in all participants. According to the results of this midpoint assessment, the intensity of the aerobic exercise and level of strengthening exercises were adjusted. The target intensity and target heart rate of the aerobic exercise were altered according to the results of the 6MWT. We increased the recommended exercise level if the participant’s 6MWT distance increased. The level of the resistance exercises was altered according to the results of the grip strength test and the 30-second chair stand test. Participants’ conditions, as a result of their exercise programs, were communicated through the app’s real-time chat service. Participant compliance with the exercise program was checked by calculating the total aerobic exercise time completed and the number of resistance exercise video clips watched.

### Measures

At baseline and at the end point of the study, we collected demographic characteristics and related medical data (ie, blood tests and body composition analyses); in addition, participants completed questionnaires for physical activity and QoL. The outcome measures for physical fitness level were performed at baseline, midintervention (ie, 6 weeks), and at the intervention end point (ie, 12 weeks).

Blood tests, completed at baseline and at the study end point, consisted of a complete blood count (ie, white blood cell count, hemoglobin, and platelets), chemistry panel (ie, albumin, cholesterol, total bilirubin, creatinine, and osteocalcin), liver function tests (ie, aspartate aminotransferase, alanine aminotransferase, alkaline phosphatase, and gamma glutamyl transferase), and a coagulation study (ie, prothrombin time [international normalized ratio]).

Data for the body composition analyses were collected using a bioelectric impedance device—Inbody 720 (Biospace). Muscle mass and body fat percentages were obtained. Body mass index (BMI) was calculated as body weight/height (kg/m^2^).

The International Physical Activity Questionnaire-Short Form (IPAQ-SF) was used to measure participants’ physical activity [15]. This questionnaire contains nine questions about the minutes per day or days per week spent doing activities of vigorous and moderate intensity and about time spent walking or sitting during the past 7 days. Using this questionnaire, we calculated the total number of metabolic equivalents (METs) per week. The calculated physical activity was classified into three groups according to the IPAQ-SF scoring system: inactive, minimally active, and highly active. The inactive group consumed less than 600 METs of physical activity, the minimally active group consumed more than 600 METs but less than 3000 METs, and the highly active group consumed a minimum of 3000 METs.

QoL related to general health was assessed by the European Organization for Research and Treatment of Cancer Quality-of-Life Questionnaire C30 (EORTC-QLQ-C30) [16]. This questionnaire contains 30 items regarding general health status, five functional scales (ie, physical, role, cognitive, emotional, and social functioning), three symptom scales (ie, fatigue, pain, and nausea or vomiting), and six single-item scales (ie, dyspnea, appetite loss, constipation, diarrhea, financial difficulties, and insomnia). Each scale includes a different set of items, which are calculated using specific coding procedures. Higher scores for the general health status and the functional scales imply positive results, whereas the symptom and the single-item scales are interpreted inversely [17].

Physical fitness was measured with the grip strength test, the 30-second chair stand test, and the 6MWT. A hand-held dynamometer—SH 5001 (Saehan Corp)—was used to assess upper-extremity muscle strength. In an upright posture with slightly abducted arm and slightly flexed elbow, the participants were instructed to hold the dynamometer with the arm and wrist both in a neutral position. After holding it for 3 seconds, maximal power was measured. This grip strength test was repeated three times and the average power was recorded [18]. Lower-limb strength was assessed by the 30-second chair stand test, which counts the maximum number of times a participant can stand up from a chair in 30 seconds. At the start, each participant was seated straight up in a chair without leaning on the backrest and with both arms folded across the chest. For 30 seconds, they repeated complete stand-up and sit-down motions as quickly as possible, and the total number of complete standing motions was counted [19]. To measure the level of cardiopulmonary endurance, the 6MWT was conducted in a 15.2-meter hallway. The total distance walked at maximal speed for 6 minutes was recorded. The physical fitness data were compared with age-specific, normal, Korean fitness values from the *National Fitness 100* project from the Korean government [20].

### Statistical Analysis

SPSS Statistics for Windows, version 24.0 (IBM Corp), was used for statistical analyses, and statistical significance was established as *P<*.05. General and clinical subject characteristics were analyzed using descriptive statistics. One-way, repeated-measures analysis of variance (ANOVA) was used to analyze changes in physical fitness over time. Because body composition, physical activity levels, and biochemical profiles were checked at baseline and after 12 weeks of the intervention, the paired *t* test or the Wilcoxon signed-rank test was used to determine the effects of the exercise intervention.

### Ethics Approval and Consent to Participate

All decisions regarding this study were approved by the Institutional Review Board of Samsung Medical Center after a complete review of clinical trial protocols (approval number: 2017-06-050). All participants provided written informed consent.

## Results

### Demographics and Clinical Characteristics

A total of 37 patients diagnosed with HCC were enrolled in this study. Among the 37 patients, 31 of them—26 (84%) males and 5 (16%) females—completed the 12-week intervention using the mHealth app program on a mobile phone with an interconnected wearable device. The 6 patients (6/37, 16%) who did not use the mHealth app and did not respond to our communications regarding the 6-week midintervention evaluation were removed from the study. The demographic and clinical characteristics of the study participants are presented in Table 1. Their mean age was 56.7 years (SD 7.7) and 84% (26/31) of them were male. Participants’ mean BMI was 25.39 kg/m^2^ (SD 3.00) and mean muscle mass was 28.98 kg (SD 5.20). Participants’ initial mean body fat percentage was 26.29% (SD 8.01), which is in the upper-normal range (normal range: 18.0%-28.0%). As for underlying CLD, 24 out of 31 (77%) patients had liver cirrhosis and 7 (23%) had chronic hepatitis. In total, 74% (23/31) of our patients had been diagnosed with HCC for more than 1 year, and 4 patients out of 31 (13%) were long-term patients of more than 5 years. In total, 19% (6/31) of patients had previously experienced hepatic decompensation, mainly variceal hemorrhage. The most common therapeutic method was surgery (17/31, 55%), followed by combination treatments (11/31, 35%) and locoregional therapies (ie, radiofrequency ablation or transarterial chemoembolization) (3/31, 10%).

**Table 1:** Demographic and clinical characteristics of participants, (N=31).

Characteristic		Value
Age (years), mean (SD)		56.7 (7.7)
**Gender, n (%)**		
	Male	26 (84)
	Female	5 (16)
Height (cm), mean (SD)		166.9 (8.2)
Weight (kg), mean (SD)		71.0 (11.3)
Body mass index (kg/m^2^), mean (SD)		25.39 (3.00)
Body fat (%), mean (SD)		26.29 (8.01)
Muscle mass (kg), mean (SD)		28.98 (5.20)
**Underlying chronic liver disease, n (%)**		
	Liver cirrhosis	24 (77)
	Chronic viral hepatitis	5 (16)
	Nonalcoholic fatty liver disease	1 (3)
	Alcoholic liver disease	1 (3)
**Diagnosis date, n (%)**		
	0-6 months ago	5 (16)
	6 months-1 year ago	3 (10)
	1-3 years ago	11 (35)
	3-5 years ago	8 (26)
	More than 5 years ago	4 (13)
**Comorbidity, n (%)**		
	Diabetes mellitus	7 (23)
	Hypertension	10 (32)
	Dyslipidemia	1 (3)
	Cardiopulmonary disease	1 (3)
**Previous experience of hepatic decompensation, n (%)**		
	None	25 (81)
	More than once	6 (19)
**Treatment, n (%)**		
	Locoregional therapies (ie, transarterial chemoembolization or radiofrequency ablation)	3 (10)
	Surgery	17 (55)
	Combination treatment	11 (35)

### Physical Fitness Measures and Compliance Rate

**Figure 3:**
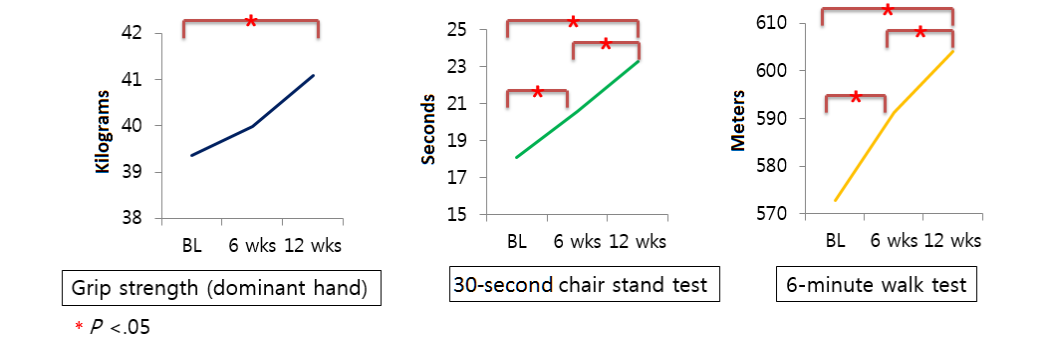
Changes in physical fitness measures. BL: baseline; wks: weeks.

Table 2 and Figure 3 present the serial changes in the objective physical fitness measures at baseline and after using the mHealth app with individually prescribed rehabilitation exercises for 6 and 12 weeks. From baseline to final measurement, grip strength was graded as poor according to the Korean national fitness normal value for 55-59-year-old males. However, compared with baseline, grip strength did improve significantly after 12 weeks of the intervention (*P*=.02). The 30-second chair stand test also showed significant improvement after 6 and 12 weeks, compared with baseline (*P*<.001 and *P*<.001, respectively), and from 6 to 12 weeks (*P*<.001). Likewise, the 6MWT showed significant improvement after 6 and 12 weeks, compared with baseline (*P*<.001 and *P*<.001, respectively), and from 6 to 12 weeks (*P*=.01).

For aerobic exercise, the stiffest decline in compliance took place at 6-7 weeks, whereas for the strengthening exercises, the most rapid decline was at 4-5 weeks (δ=9.68% and δ=11.29%, respectively).

**Table 2:** Changes in physical fitness measures.

Measure	Baseline, mean (SD)	6 weeks, mean (SD)	12 weeks, mean (SD)	Baseline vs 6 weeks, *P* value	6 weeks vs 12 weeks, *P* value	Baseline vs 12 weeks, *P* value
Grip strength (kg)	39.35 (9.98)	39.98 (9.69)	41.10 (10.52)	.69	.31	.02
30-second chair stand test (seconds)	18.10 (3.29)	20.55 (3.03)	23.26 (3.79)	<.001	<.001	<.001
6-minute walk test (meters)	572.90 (49.15)	591.19 (49.61)	604.07 (51.59)	<.001	.01	<.001

### Body Composition and Self-Reported Physical Activity

**Figure 4:**
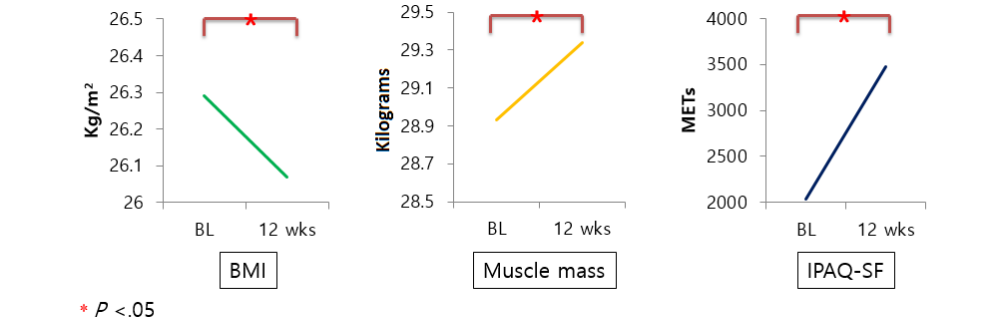
Changes in body composition and self-reported physical activity. BL: baseline; BMI: body mass index; IPAQ-SF: International Physical Activity Questionnaire-Short Form; METs: metabolic equivalents.

As shown in Table 3 and Figure 4, muscle mass increased significantly after 12 weeks of prescribed exercise (*P*=.03). The change in BMI was insignificant, and body fat percentage declined but without statistical significance (*P*=.08 and *P*=.51, respectively). At baseline, the mean, weekly, physical activity score reported on the IPAQ-SF was 2031.95 (SD 2236.60), placing participants in the minimally active group. After completing 12 weeks of the exercise intervention, the mean IPAQ-SF score increased to 3479.71 (SD 2640.08), which places participants in the highly active group. The increase in the IPAQ-SF score was significant after 12 weeks of the intervention (*P*=.01) (see Table 3 and Figure 4).

**Table 3:** Changes in body composition and self-reported physical activity.

Measure	Baseline, mean (SD)	12 weeks, mean (SD)	*P* value
Body mass index (kg/m^2^)	25.39 (3.00)	25.57 (3.08)	.08
Body fat (%)	26.29 (8.01)	26.07 (7.86)	.51
Muscle mass (kg)	28.98 (5.15)	29.34 (5.31)	.03
IPAQ-SF^a^ (METs^b^)	2031.95 (2236.60)	3479.71 (2640.08)	.01

^a^IPAQ-SF: International Physical Activity Questionnaire-Short Form.

^b^METs: metabolic equivalents.

### Biochemical Profile and Complications

Serum levels of aspartate aminotransferase, alanine aminotransferase, alkaline phosphatase, and gamma glutamyl transferase, which indicate liver function, showed no significant change after 12 weeks of exercise. Insignificant results were seen in albumin and creatinine, which are synthesized in liver cells, and in total bilirubin and prothrombin time (ie, international normalized ratio). No significant difference was observed in osteocalcin, which is inversely related to nonalcoholic fatty liver disease (NAFLD); cholesterol, which increases in NAFLD; or complete blood count. During 12 weeks of exercise, no complications were reported through the in-app chat service, including hepatic decompensation in the form of variceal hemorrhage, ascites, jaundice, or hepatic encephalopathy.

### Quality of Life

Table 4 presents the changes in QoL after 12 weeks of monitored, individualized exercise via our mHealth app. Based on the EORTC-QLQ-C30, the most common symptom was fatigue, followed by dyspnea and insomnia. After 12 weeks of individually prescribed exercise through the mHealth app, all symptoms improved insignificantly, except pain, which decreased significantly (*P*=.04). Global health status and all functional scales trended toward improvement after 12 weeks of the intervention, though without statistical significance.

**Table 4:** Changes in quality of life.

EORTC-QLQ-C30^a^ item		Score at baseline, mean (SD)	Score at 12 weeks, mean (SD)	*P* value
Global health status and quality of life^b^		72.50 (17.52)	74.44 (17.63)	.43
**Functional scale ^b^**				
	Physical functioning	85.77 (10.31)	87.55 (11.30)	.43
	Role functioning	87.22 (13.62)	89.44 (16.65)	.38
	Emotional functioning	84.16 (18.48)	87.50 (12.90)	.28
	Cognitive functioning	80.55 (13.19)	86.11 (11.64)	.06
	Social functioning	85.55 (24.65)	93.33 (12.06)	.08
**Symptom scale or single item^c^**				
	Fatigue	25.18 (12.69)	23.33 (15.25)	.48
	Nausea and vomiting	2.22 (5.76)	2.17 (6.31)	.66
	Pain	10.55 (14.17)	6.11 (11.14)	.04
	Dyspnea	22.22 (26.74)	17.77 (24.34)	.35
	Insomnia	15.55 (22.71)	14.44 (20.86)	.80
	Appetite loss	6.66 (16.14)	5.55 (12.63)	.71
	Constipation	12.00 (17.83)	11.11 (15.98)	.80
	Diarrhea	12.00 (17.83)	11.11 (15.98)	.71
	Financial difficulties	13.33 (25.67)	7.77 (16.80)	.20

^a^EORTC-QLQ-C30: European Organization for Research and Treatment of Cancer Quality-of-Life Questionnaire C30.

^b^Higher scores imply positive results.

^c^Lower scores imply positive results.

## Discussion

### Principal Findings

In this study, we found that it was safe and effective for compensated HCC patients who have completed anticancer therapy to undergo 12 weeks of individually prescribed rehabilitation exercises using our mHealth app and interconnected IoT wearable device. Our surveillance system, composed of the wearable IoT device and real-time communication chat service with health care professionals, found no complications or biochemical deterioration during the 12 weeks of the intervention. Compared to baseline, statistically significant improvements were found in the physical fitness measures (ie, grip strength, 30-second chair stand test, and 6MWT), body composition (ie, muscle mass), self-reported amount of physical activity (ie, IPAQ-SF), and pain. All symptoms trended toward improvement in the QoL scales (ie, EORTC-QLQ C30) after 12 weeks of the intervention. Compliance with the aerobic and strengthening exercises decreased slightly but was maintained overall throughout the intervention period. To the best of our knowledge, this was the first study to use an mHealth app and interconnected IoT device for individually prescribed rehabilitation exercises in compensated HCC patients after anticancer treatment.

Growing interest had led to many studies investigating the association of cancer and exercise. Increased risks for several solid cancers, including breast, endometrial, prostate, and colorectal, were inversely associated with physical activity [21]. Recent studies indicate that even after diagnosis with those solid cancers, regular exercise resulted in more survival years and a lower tumor recurrence rate [22-24]. The biochemical mechanisms of such findings are still being discovered, but exercise-dependent regulation of the tumor microenvironment is currently held to be the primary mechanism. In experimental models, chronic exposure to exercise stimulated interorgan signaling through the complex control of hormones, cytokines, and growth factors. Consequently, reprogramming of the systemic milieu was stimulated, resulting in regulation of the tumor microenvironment through angiogenesis, immune regulation, and metabolism [25].

The potential benefit of exercise on liver disease was first studied in NAFLD, of which the key pathophysiological mechanism is insulin resistance [26]. In terms of HCC, a Taiwanese cohort study [27], which was later confirmed by the National Institutes of Health [28], found that a degree of physical activity correlated with a decline in HCC risk. The antitumoral effect of exercise on HCC is mainly associated with decreases in body weight, insulin resistance, and chronic inflammation [29]. In a previous experimental study with a rodent model of nonalcoholic steatohepatitis, regular exercise inhibited HCC development by stimulating adenosine monophosphate-activated protein kinase and inhibiting mammalian target of rapamycin complex 1 [7].

However, only a few studies have considered the potential therapeutic effects of exercise after the development of HCC. One Japanese study had HCC patients work out at their anaerobic thresholds from 1 month preoperation until 6 months postoperation. The exercise group showed decreased whole-body mass, fat mass, and fasting serum insulin; they even showed improvement in the anaerobic threshold, peak oxygen consumption, and insulin resistance [30]. Contrary to our intervention period of 12 weeks, the Japanese study’s program began before hepatectomy and lasted for 6 months postoperation. Also, that study’s exercise intervention was targeted to the anaerobic threshold and consisted only of stretching and walking, whereas our study’s exercise protocol combined stretching, aerobic, and muscle-strengthening exercises. Both studies adjusted the exercise program during the intervention. Most importantly, compared to the Japanese patients who visited the study center three times a week for 60-minute exercise sessions with an exercise trainer, our participants exercised freely outside the hospital via the mHealth program. In our study, exercise compliance and safety were monitored via the mHealth devices.

Another study reported that a median of 13 days of in-hospital exercise among HCC patients with underlying CLD did not worsen the Child-Pugh class, maintained the 6MWT distance, and significantly improved heart rate variability [6]. Compared to the thorough exercise program for upper and lower extremities in our study protocol, the exercise program of that previous study contained stretching, strengthening, and balance training for only the lower extremities. Unlike our study, the exercises in the previous study were not tailored according to individual fitness levels nor adjusted during the intervention period. Also, those exercises were initiated 1 day after anticancer treatment for HCC. Though the previous study evaluated 6MWT and biochemical profiles by blood sampling, the relatively short exercise period (ie, median 7.5 days) of that in-hospital study was not long enough to show physiological or biological changes.

Contrary to malignancies that occur without any underlying condition, HCC commonly arises in cirrhotic liver or viral hepatitis. Most HCC patients suffer from CLD for several years before developing HCC, so their QoL is low and worsens after cancer treatment. One global survey study found that among HCC patients, 81% receiving sorafenib (ie, tyrosine kinase inhibitor), 45% receiving selective internal radiation therapy, and 32% receiving transarterial chemoembolization reported impaired QoL [31]. Our unpublished previous survey study also found that CLD patients, including those with HCC, reported a low level of health-related QoL and physical activity, being minimally active. Boosting the low physical fitness levels of chronically ill HCC patients is a complex process, involving both motivating and safely leading these patients to exercise properly.

As the chronicity of the underlying CLD in HCC patients deteriorates their general physical activity levels, we used an mHealth app via a mobile phone and interconnected wearable IoT device. This system allowed close monitoring of patients’ vital signs and exercise compliance, which allowed all participants to complete the 12-week exercise program without any complications or biochemical deterioration. Despite increasing interest and studies on mHealth for cancer patients, studies involving the use of IoT devices among cancer patients are scarce. A few studies using wearable IoT devices and mHealth apps were conducted to promote physical activity in breast cancer survivors and childhood cancer survivors [32-34]. Only two studies used an IoT device among cancer patients under treatment: one is our previous study involving colorectal cancer patients receiving chemotherapy, and the other study estimated the symptom severity of chemotherapy with a wearable IoT device in gastrointestinal cancer patients [4,35]. We are the first to apply mHealth with an IoT device to HCC patients. According to the satisfaction survey after 12 weeks of our mHealth program for HCC patients, 84% of participants reported medium-to-high satisfaction with the mHealth program. A total of 87% of participants wanted to continue to use the program after the study ended.

This study has several limitations. First, the study population was small and the intervention period was short. In most of the previously conducted feasibility or pilot studies regarding new protocols of exercise interventions among a cancer population, 10-30 patients were enrolled [36-39]. Since this was the first study to apply an IoT-based individualized exercise program among compensated HCC patients who had completed therapy, we considered that the final participant number of 31 was sufficient. The intervention period was selected based on previous interventional studies using mobile apps among cancer patients. Most studies were conducted for 12 weeks or 3 months [11,18,40]. Further studies with large sample sizes and longitudinal intervention periods will support the results of this study. Second, the completion rate of this study was low. The completion rate in exercise intervention studies of cancer populations differs based on the traits of the population, such as dominant gender, mean age, severity of cancer, general condition, needs of exercise, and type of ongoing anticancer therapy. For example, one study that compared the physical fitness effect of mHealth and conventional exercise using a brochure among breast cancer patients—all female patients, mean age 50.3 years, and 12-week follow-up—showed a 95.2% completion rate [18]. In contrast, 73.9% of participants diagnosed with prostate cancer—all male patients, mean age 68.4 years, and 12-week follow-up—completed the 12-week course of a home-based *exergaming* intervention [41]. The completion rate of 84% (31/37) in this study—84% male patients, mean age 56.7 years, and 12-week follow-up—is not lower than that of the previous prostate cancer exercise intervention. Also, without a previous study of a 12-week exercise intervention among HCC patients, it is difficult to confirm that the completion rate of our study is low. Third, we used no control group in this study. Inclusion of a sex- and age-matched control group could have strengthened our results. However, as this was the first study to apply an mHealth-based exercise program to HCC patients, comparing the outcome measures of the initial, 6-week, and 12-week visits of the mHealth exercise group without a control group yielded significant results. Finally, because this study was conducted among compensated HCC patients after cancer therapy, it would be inappropriate to generalize these results. Further studies with a larger sample size and a randomized controlled study involving HCC patients with diverse medical statuses are needed.

### Conclusions

In this study, compensated HCC patients after therapy underwent 12 weeks of comprehensive cancer care through an mHealth app. The app provided general medical information on HCC, nutritional information, real-time communication with a health care provider, and an individually prescribed rehabilitation exercise program. The exercise program was monitored through an IoT device. The intervention significantly improved physical fitness, body composition, and self-reported physical activity without any complications or biochemical deterioration. We found it safe and effective for compensated HCC patients after cancer treatment to exercise using the mobile app and interconnected IoT wearable device. This advanced technology allows effective and practical, patient-centered, supervised home exercise therapy to be an alternative to conventional, unfeasible, hospital-oriented exercise programs.

## Abbreviations

6MWT: 6-minute walk test
ANOVA: analysis of variance
BMI: body mass index
CLD: chronic liver disease
EORTC-QLQ-C30: European Organization for Research and Treatment of Cancer Quality-of-Life Questionnaire C30
HCC: hepatocellular carcinoma
IoT: Internet of Things
IPAQ-SF: International Physical Activity Questionnaire-Short Form
METs: metabolic equivalents
mHealth: mobile health
mUICC: modified Union for International Cancer Control
NAFLD: nonalcoholic fatty liver disease
QoL: quality of life
